# Deadly Emanations

**Published:** 1859-08

**Authors:** 


### DEADLY EMANATIONS.

Persons descending wells, or caves, or vaults, die speedily, not from any poisonous effect in the atmosphere of those places, they die because there is little or no oxygen in it, no nutriment for the lungs and blood; it is upon the same principle that a candle dies out if let down into such an atmosphere, the flame getting less and less bright, "burning blue" in proportion as the supply of oxygen is in course of exhaustion. It is in this connection, that vulgar minds have associated ghosts, and apparitions, and death, with a blue flame, whether in the candle, or in the fire place.

Whether there is this innutritous air in a well, or cave, or vault, should be always previously determined, either by letting a candle down, or setting paper, or shavings on fire, and throwing them in.

If from neglecting these precautions a person faints away, the first best thing to do, while preparations are being made for removal, is to dash down buckets of cold water, this carries some oxygen, some pure air with it; it also absorbs some of the deadly gas, and in the third place, by cooling the locality, the heaviest and most destructive gas condenses and falls to the surface, rests on the ground, thus allowing a purer air to take its place.

